# Psychological symptoms are associated with screen and exercise time: a cross-sectional study of Chinese adolescents

**DOI:** 10.1186/s12889-020-09819-7

**Published:** 2020-11-12

**Authors:** Feng Zhang, Xiaojian Yin, Cunjian Bi, Liu Ji, Huipan Wu, Yuqiang Li, Yi Sun, Sien Ren, Guodong Wang, Xiaofang Yang, Ming Li, Yuan Liu, Ge Song

**Affiliations:** 1 Key Laboratory of Adolescent Health Assessment and Exercise Intervention of Ministry of Education, East China Normal University, Shanghai, 200241 China; 2 College of Physical Education and Health, East China Normal University, Shanghai, 200241 China; 3 College of Economics and Management, Shanghai Institute of Technology, Shanghai, 201418 China

**Keywords:** Mental disorders, Mental health, Screen time, Exercise time, Psychological symptoms, Chinese adolescents

## Abstract

**Background:**

Mental disorders among adolescents have become a worldwide public health problem. This study aimed to estimate the best combination of exercise time and screen time to promote the mental health of Chinese adolescents.

**Methods:**

Participants included 7200 Chinese adolescents aged 13–18 years from six regions of China. Screen and exercise time data were collected via a self-rated questionnaire. Psychological symptoms (e.g., often feeling depressed, school-weary) were evaluated with the “Multidimensional Sub-health Questionnaire of Adolescents”. Chi-squared tests and logistic regression analysis were conducted to investigate the differences in and correlations among screen time, exercise time, and psychological symptoms.

**Results:**

The overall detection rate of psychological symptoms among Chinese adolescents was 21.4% (22.1% for boys and 20.6% for girls). Psychological symptom detection rates were lowest among adolescents with 1–2 h/d of screen time (19.5%) and those with 30–60 min/d of exercise time (17.3%). Screen time > 2 h/d (OR = 1.38, *P* < 0.001) and exercise time < 30 min/d (OR = 1.62, *P* < 0.001) were positively correlated with psychological symptoms.

**Conclusion:**

Screen and exercise time are associated with psychological symptoms in Chinese adolescents. A combination of 1–2 h/d of screen time and 30–60 min/d of exercise time is provided as a reference for better mental health.

## Background

Involving profound individual biological, social, and psychological changes, adolescence is a difficult stage of life in which to change health-related behaviours, with many social interactions being impacted [1]. In addition, from a mental health perspective, it is important to provide mental health services during this time because adolescence is a critical period in the development of mental disorders. Mental disorders (e.g., depression or anxiety) are associated with other chronic diseases, further increasing their morbidity and mortality [2, 3], and they have become a serious global public health issue among adolescents. Unfortunately, mental disorders affect 10–20% of adolescents worldwide [4–6]. Chinese adolescents’ mental health has also deteriorated across birth cohorts since the early 1990s [7].

It is well known that economic and technological improvements increase screen time among young students [8–10]. In China, weekend screen time was reported to be > 2 h/d for 41.5% of adolescents [11]. Recent research has shown that screen-based sedentary behaviour is detrimental to young people’s mental health [12]. A study by Fu et al. [13] found that a screen time > 2 h/d among middle school students was associated with psychological symptoms and self-injury. However, the American Academy of Pediatrics rescinded the guideline that screen time should be limited to two hours because they realized that screen time is an essential part of all adolescents’ lives. A growing body of research conducted over the past decade suggests that a certain amount of time spent online (i.e., < 2 h/d) can actually benefit young people [14, 15]. A review of 36 studies published between 2002 and 2017 also indicated that teens use digital communication to enhance relationships by sharing intimacy, displaying affection, and arranging meet-ups and activities [16]. In addition to screen time, exercise time may be another factor related to mental health [17]. The World Health Organization recommended that children and youth aged 5–17 should engage in at least 60 min of moderate- to vigorous-intensity physical activity daily for physical and psychological health benefits [18]. A prospective study suggested that if adolescents performed physical exercise for ≥1 h each week, their risk of suffering from depression would be reduced by 8% [19]. A cross-sectional study involving 1.2 million adolescents between 2011 and 2015 concluded that 30–60 min of exercise 3–5 days per week could result in better mental health [20]. However, an exercise time > 90 min/d may have a negative impact on mental health because of excessive exercise or fatigue [20]. The relationship between screen time, exercise time, and mental health requires further investigation and verification.

Several analyses have suggested that promoting physical activity (including exercise) and decreasing screen time might improve mental health in children and adolescents [21–23]. There is a growing view that screen time and physical inactivity interact to increase psychological problems [24, 25]. Given the high prevalence of physical inactivity and screen-based sedentary behaviours worldwide [26] and in China [27], the present study aimed to determine the best combination of exercise time and screen time to promote the mental health of Chinese adolescents and provide a reference for screen time and exercise time to prevent mental disorders in Chinese adolescents.

## Methods

### Data source and participants

**Fig. 1:**
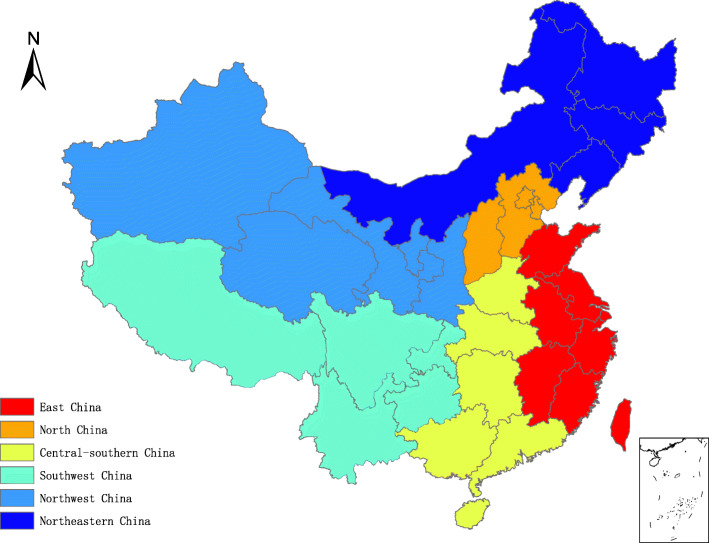
Map of six geographical regions of China created by ArcGIS ArcMap 10.5 of ESRI. Note: ESRI stand for Environmental Systems Research Institute, Inc., Redlands, California, USA

Data for this study were obtained from a major project of the Ministry of Education named “Preparation of New Evaluation Methods and Criteria for Physical Health of Children and Adolescents in China” (No. 11001–412221-15017). This project aimed to develop new evaluation methods and criteria regarding the physical health of Chinese children and adolescents aged 7–18 years and was conducted by the Key Laboratory of Adolescent Health Assessment and Exercise Intervention of the Ministry of Education in 2015–2016. A nationally representative survey was conducted in 27 of 31 provinces, which were evenly distributed across six geographical regions of China (East China, North China, Central-South China, Northwest China, Southwest China, and Northeast China) (Fig. 1). This project was approved by the Ethics Committee for Human Experiments of East China Normal University (Grant No. HR006–2019). After approval by schools, participants and their parents were informed about the purpose of the study, and written informed consent was obtained from all participants. All students’ names were digitally coded to avoid compromising their personal information.

**Fig. 2:**
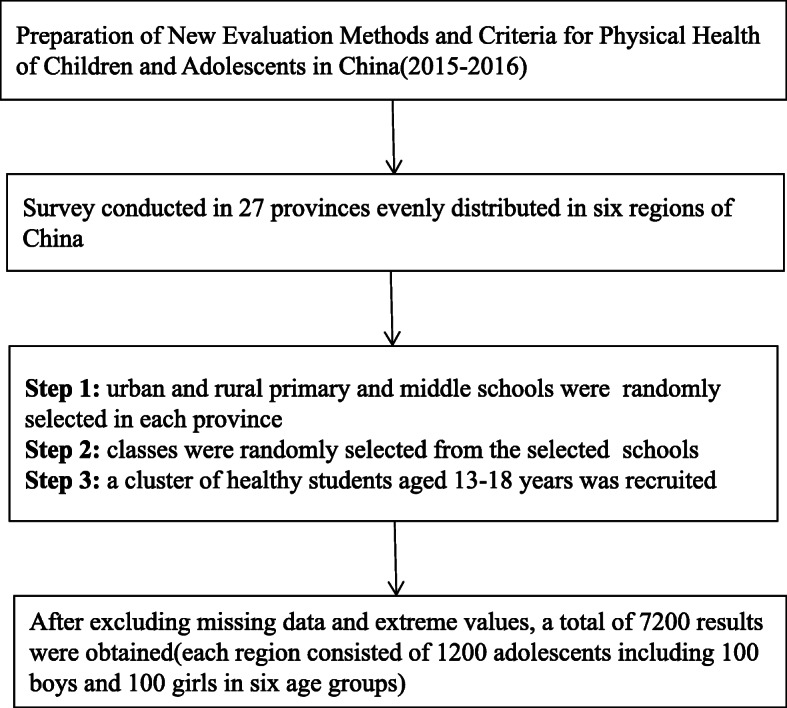
Flow chart of data from the “Preparation of New Evaluation Methods and Criteria for Physical Health of Children and Adolescents in China”

Stratified randomized cluster sampling was used to recruit participants to ensure the representativeness of the sample. First, considering socioeconomic factors, urban and rural schools in each province were randomly selected. Classes were randomly recruited from the selected schools. Subsequently, clusters of students in the selected classes who did not have physical or mental disabilities and who agreed to participate in the investigation were recruited from the selected classes as participants (Fig. 2). Finally, a total of 90,031 participants were selected for this project.

Questionnaires with a completion rate of lower than 90% were regarded as having missing data and were excluded. Finally, 80,792 valid responses were obtained from Chinese children and adolescents aged 7–18 years old. We extracted 200 responses (including responses from 100 girls and 100 boys) from each of the six age groups (13–18 years old) from each of the six geographical regions of China. Finally, 7200 results were obtained for the present study.

### Instruments

A self-administered questionnaire collecting demographic information and data on psychological symptoms, screen time, and exercise time was completed by the adolescents independently over a 40-min period in the presence of graduate students majoring in human sports science in classrooms after school.

Psychological symptoms were assessed using the Multidimensional Sub-health Questionnaire of Adolescents (MSQA) [28], which was designed specifically for assessing the psychological symptoms of adolescents and has been applied in several studies [22, 29–31]. The validity and reliability of the MSQA have been confirmed [32], and the Cronbach’s alpha (α) coefficient was 0.96 [22]. The responsiveness of the MSQA has been shown to be relatively good and was confirmed in a large sample [33]. The MSQA is a self-reported screening tool to investigate uncomfortable symptoms experienced by respondents during the last 3 months and comprises 39 items in three dimensions: emotional symptoms, behavioural symptoms, and social adaptation difficulties. Emotional symptoms are evaluated with 17 items (e.g., “often feeling depressed”), behavioural symptoms are assessed with nine items (e.g., “often having an impulse to smash things”), and social adaptation difficulties are measured with 13 items (e.g., “always hating school”). Each item has six response options. Participants choose the option that best describes the duration for which they experienced each symptom over the reference period: “never or less than 1 week”, “more than 1 week”, “more than 2 weeks”, “more than 1 month”, “more than 2 months”, and “more than 3 months”. For the present data analysis, the responses were reclassified, with “more than 1 month,” “more than 2 months,” and “more than 3 months” recorded as 1 and the remainder recorded as 0. Scores for the three dimensions (emotional symptoms, behavioural symptoms, and social adaptation difficulties) were derived by summing the scores for the corresponding items. Psychological symptoms were calculated by summing the scores of the 39 items. As stipulated in the MSQA National Norm Development [28], the 90th percentile was taken as the cut-off value for psychological symptoms for adolescents of all ages. The cut-off scores for emotional symptoms, behavioural symptoms, social adaptation difficulties, and psychological symptoms were ≥ 3, ≥1, ≥4, and ≥ 8, respectively.

Data on screen time were obtained using the following question: How many hours per day do you spend playing video games (including on the computer or mobile phone) and watching TV/video programmes? All the time spent on screen-based electronic mediums and devices, such as chatting online, reading the latest news online, and participating in online courses, were included. There were four options from which the adolescents could choose: “less than 1 hour”, “1–2 h”, “2–3 h”, and “more than 3 hours”.

Data on exercise time were also obtained by self-reported questionnaires, the most commonly used method of exercise assessment [34]. Unlike physical activity more broadly, exercise time is only a subset of physical activity that is planned, structured, and repetitive and has as a final or an intermediate objective, i.e., the improvement or maintenance of physical fitness [35]. The participants reported exercise time using the following question: “How many hours per day do you spend exercising besides in PE class (such as running, swimming, and playing all kinds of ball sports)?” The responses included “less than 30 minutes”, “30–60 min”, “1–2 h”, and “more than 2 hours.”

### Statistical analysis

Age is expressed as the mean ± standard deviation. The detection rates of adolescents’ emotional symptoms, behavioural symptoms, social adaptation problems, and psychological symptoms were compared according to the screen and exercise time categories using chi-squared tests. With psychological symptoms as the primary outcome variable and its three dimensions as another level of the outcome variable, binary logistic regression analysis was conducted to estimate the relationships among screen time, exercise time, and psychological symptoms. Since weight status has been shown to be associated with adolescents’ screen time [36], physical activity [37], [38] and mental health [25], age, weight, and BMI were controlled in the binary logistic regression analysis to eliminate these effects. With participants with a screen time of 1–2 h/d and those with an exercise time of 30–60 min/d as the reference groups, we also conducted an interaction of screen time and exercise time on psychological symptoms after controlling for sex, age, weight, and BMI to determine the best combination of exercise time and screen time to promote the mental health of Chinese adolescents. The test level was α = 0.05. All analyses were conducted with SPSS software version 23.0 (SPSS Inc., Chicago, IL, USA).

## Results

The results showed that the average age of the participating adolescents was 15.50 ± 1.71 years. More girls had screen times < 1 h/d or 1–2 h/d than boys, but more boys had screen times > 2 h/d. In addition, more boys had exercise times of 30–60 min/d or > 60 min/d than girls. The detection rate of psychological symptoms was 21.4% (22.1% for boys and 20.6% for girls). There was no significant difference in the detection rate of psychological symptoms between boys and girls. The detection rates of emotional symptoms, behavioural symptoms, and social adaptation difficulties were higher among boys than girls (Table 1).

**Table 1:** Screen time, exercise time, and psychological symptoms of Chinese adolescents

Items	Boys	Girls	Total
Sample size	3600	3600	7200
Age(years)	15.50 ± 1.71	15.50 ± 1.71	15.50 ± 1.71
Screen time (h/d), N (%)			
<1	1792 (49.8)	1906 (52.9)	3698 (51.4)
1–2	1108 (30.8)	1186 (32.9)	2294 (31.9)
>2	700 (19.4)	508 (14.1)	1208 (16.8)
Exercise time (min/d), N (%)			
<30	1311 (36.2)	2044 (56.8)	3355 (46.6)
30–60	1551 (43.1)	1270 (35.3)	2821 (39.2)
>60	738 (20.5)	286 (7.90)	1024 (14.2)
Psychological symptoms, N (%) (%)			
Emotional symptoms	1001 (27.8)	994 (27.6)	1995 (27.7)
Behavioral symptoms	1021 (28.4)	955 (26.5)	1976 (27.4)
Social adaptation difficulties	656 (18.2)	577 (16.0)	1233 (17.1)
Psychological symptoms	797 (22.1)	743 (20.6)	1540 (21.4)

Table 2 compares the psychological symptom detection rates among Chinese adolescents by screen and exercise time. In general, the detection rates of emotional symptoms, behavioural symptoms, social adaptation difficulties, and psychological symptoms were the highest among adolescents with a screen time > 2 h/d, and the difference was significant (χ^2^ = 14.34, χ^2^ = 28.11, χ^2^ = 17.31, and χ^2^ = 14.31, respectively; *P* < 0.01). The detection rates of the three dimensions and psychological symptoms were lowest among those with an exercise time of 30–60 min/d, followed by those with an exercise time > 60 min/d or < 30 min/d (χ^2^ = 74.89, χ^2^ = 25.34, χ^2^ = 56.58, and χ^2^ = 59.22, respectively; P < 0.01).

**Table 2:** Comparisons of psychological symptom detection rates among Chinese adolescents by screen and exercise time (%)

Sex	Category	Group	N	Emotional symptoms			Behavioral symptoms			Social adaption difficulties			Psychological symptoms		
				N (%)	***χ***^**2**^	***P***	N (%)	***χ***^**2**^	***P***	N (%)	***χ***^**2**^	***P***	N (%)	***χ***^**2**^	***P***
Boys	Screen time (h/d)	<1	1792	519 (29.0)	7.69	0.02	488 (27.2)	19.37	< 0.001	325 (18.1)	9.68	0.01	397 (22.2)	6.63	0.04
		1–2	1108	274 (24.7)			288 (26.0)			178 (16.1)			223 (20.1)		
		>2	700	208 (29.7)			245 (35.0)			153 (21.9)			177 (25.3)		
	Exercise time (min/d)	<30	1311	450 (34.3)	43.66	< 0.001	414 (31.6)	10.90	< 0.001	301 (23.0)	31.21	< 0.001	354 (27.0)	28.29	< 0.001
		30–60	1551	374 (24.1)			405 (26.1)			244 (15.7)			300 (19.3)		
		>60	738	177 (24.0)			202 (27.4)			111 (15.0)			143 (19.4)		
Girls	Screen time (h/d)	<1	1906	521 (27.3)	9.78	0.01	521 (27.3)	10.26	0.01	316 (16.6)	6.76	0.03	394 (20.7)	7.11	0.03
		1–2	1186	305 (25.7)			279 (23.5)			166 (14.0)			224 (18.9)		
		>2	508	168 (33.1)			155 (30.5)			95 (18.7)			125 (24.6)		
	Exercise time (min/d)	<30	2044	640 (31.3)	41.78	< 0.001	593 (29.0)	20.23	< 0.001	392 (19.2)	38.72	< 0.001	493 (24.1)	42.58	< 0.001
		30–60	1270	268 (21.1)			280 (22.0)			140 (11.0)			187 (14.7)		
		>60	286	86 (30.1)			82 (28.7)			45 (15.7)			63 (22.0)		
Total	Screen time (h/d)	<1	3698	1040 (28.1)	14.34	< 0.001	1009 (27.3)	28.11	< 0.001	641 (17.3)	17.31	< 0.001	791 (21.4)	14.31	< 0.001
		1–2	2294	579 (25.2)			567 (24.7)			344 (15.0)			447 (19.5)		
		>2	1208	376 (31.1)			400 (33.1)			248 (20.5)			302 (25.0)		
	Exercise time (min/d)	<30	3355	1090 (32.5)	74.89	< 0.001	1007 (30.0)	25.34	< 0.001	693 (20.7)	56.58	< 0.001	847 (25.2)	59.22	< 0.001
		30–60	2821	642 (22.8)			685 (24.3)			384 (13.6)			487 (17.3)		
		>60	1024	263 (25.7)			284 (27.7)			156 (15.2)			206 (20.1)		

Among boys, the detection rates of emotional symptoms, behavioural symptoms, social adaptation difficulties, and psychological symptoms were lowest in those with a screen time of 1–2 h/d, followed by those with a screen time < 1 h/d. The detection rates of psychological symptoms, emotional symptoms, behavioural symptoms, and social adaptation difficulties were highest among boys with a screen time > 2 h/d (χ^2^ = 7.69, χ^2^ = 19.37, χ^2^ = 9.68, and χ^2^ = 6.63, respectively; *P* < 0.05). The detection rates of emotional symptoms, behavioural symptoms, social adaptation difficulties, and psychological symptoms among boys with an exercise time < 30 min/d were significantly higher than those among boys with an exercise time of 30–60 min/d or > 60 min/d (χ^2^ = 43.66, χ^2^ = 10.90, χ^2^ = 31.21, χ^2^ = 28.29, respectively, *P* < 0.001)(Table 2).

Among girls, the detection rates of emotional symptoms, behavioural symptoms, social adaptation difficulties, and psychological symptoms were the lowest in those with a screen time of 1–2 h/d, followed by those with a screen time < 1 h/d or > 2 h/d, (χ^2^ = 9.78, χ^2^ = 10.26, χ^2^ = 6.76, and χ^2^ = 7.11, respectively; *P* < 0.05). The detection rates of emotional symptoms, behavioural symptoms, social adaptation difficulties, and psychological symptoms were lowest among girls with an exercise time of 30–60 min/d, followed by those with an exercise time > 60 min/d or < 30 min/d (χ^2^ = 41.78, χ^2^ = 20.23, χ^2^ = 38.72, and χ^2^ = 42.58, respectively; *P* < 0.001) (Table 2).

After age and body mass index (BMI) were controlled, binary logistic regression analyses were conducted, with those with a screen time of 1–2 h/d and those with an exercise time of 30–60 min/d as the reference groups. The results are shown in Table 3. In general, compared with adolescents with an exercise time of 30–60 min/d, adolescents with an exercise time < 30 min/d had 1.63 times as many emotional symptoms (*P* < 0.001), 1.34 times as many behavioural symptoms (*P* < 0.001), 1.65 times as many social adaptation difficulties (*P* < 0.001), and 1.62 times as many psychological symptoms (*P* < 0.001). In addition, adolescents with a screen time > 2 h/d had 1.34 times as many emotional symptoms, 1.51 times as many behavioural symptoms, 1.46 times as many social adaptation difficulties, and 1.38 times as many psychological symptoms as those with a screen time of 1–2 h/d (*P* < 0.001). The risks for psychological symptoms among girls with an exercise time < 30 min/d or > 60 min/d were 1.84 times (*P* < 0.001) and 1.64 times (*P* < 0.001) higher than that for girls with an exercise time of 30–60 min/d. Therefore, exercise times < 30 min/d and > 60 min/d were risk factors for psychological symptoms among girls. The risk for psychological symptoms among boys with an exercise time < 30 min/d was 1.54 times higher than that for boys with an exercise time of 30–60 min/d (P < 0.001) (Table 3).

**Table 3:** Binary logistic regression analysis of psychological symptoms of Chinese adolescents by screen and exercise time

Gender	Variable	Group	Emotional symptoms		Behavioral symptoms		Social adaption difficulties		Psychological symptoms	
			***OR***(95%***CI***)	***P***	***OR***(95%***CI***)	***P*** value	***OR***(95%***CI***)	***P*** value	***OR***(95%***CI***)	***P***
Boys	Scree time (h/d)	<1	1.24 (1.05–1.47)	0.01	1.07 (0.90–1.26)	0.46	1.16 (0.95–1.41)	0.15	1.13 (0.94–1.36)	0.20
		1–2	1		1		1		1	
		>2	1.29 (1.04–1.59)	0.02	1.53 (1.25–1.88)	< 0.001	1.46 (1.15–1.86)	< 0.001	1.34 (1.07–1.68)	0.01
	Exercise time (min/d)	<30	1.65 (1.40–1.94)	< 0.001	1.31 (1.11–1.54)	< 0.001	1.60 (1.32–1.93)	< 0.001	1.54 (1.29–1.84)	< 0.001
		30–60	1		1		1		1	
		>60	0.99 (0.81–1.22)	0.95	1.07 (0.88–1.30)	0.52	0.95 (0.74–1.21)	0.67	1.00 (0.80–1.25)	0.98
Girls	Scree time (h/d)	<1	1.09 (0.92–1.28)	0.32	1.22 (1.03–1.45)	0.02	1.22 (1.00–1.50)	0.05	1.12 (0.93–1.34)	0.23
		1–2	1		1		1		1	
		>2	1.43 (1.14–1.79)	< 0.001	1.43 (1.13–1.80)	< 0.001	1.41 (1.07–1.86)	0.01	1.40 (1.09–1.80)	0.01
	Exercise time (min/d)	<30	1.70 (1.45–2.01)	< 0.001	1.45 (1.23–1.70)	< 0.001	1.92 (1.56–2.36)	< 0.001	1.84 (1.53–2.22)	< 0.001
		30–60	1		1		1		1	
		>60	1.61 (1.21–2.14)	< 0.001	1.42 (1.07–1.90)	0.02	1.51 (1.05–2.17)	0.03	1.64 (1.19–2.25)	< 0.001
Total	Scree time (h/d)	<1	1.16 (1.03–1.31)	0.02	1.14 (1.01–1.29)	0.03	1.19 (1.03–1.37)	0.02	1.12 (0.99–1.28)	0.08
		1–2	1		1		1		1	
		>2	1.34 (1.15–1.56)	< 0.001	1.51 (1.29–1.76)	< 0.001	1.46 (1.22–1.75)	< 0.001	1.38 (1.17–1.63)	< 0.001
	Exercise time (min/d)	<30	1.63 (1.46–1.83)	< 0.001	1.34 (1.19–1.50)	< 0.001	1.65 (1.44–1.89)	< 0.001	1.62 (1.43–1.83)	< 0.001
		30–60	1		1		1		1	
		>60	1.17 (0.99–1.38)	0.06	1.20 (1.02–1.41)	0.03	1.14 (0.93–1.40)	0.20	1.21 (1.01–1.45)	0.04

**Fig. 3:**
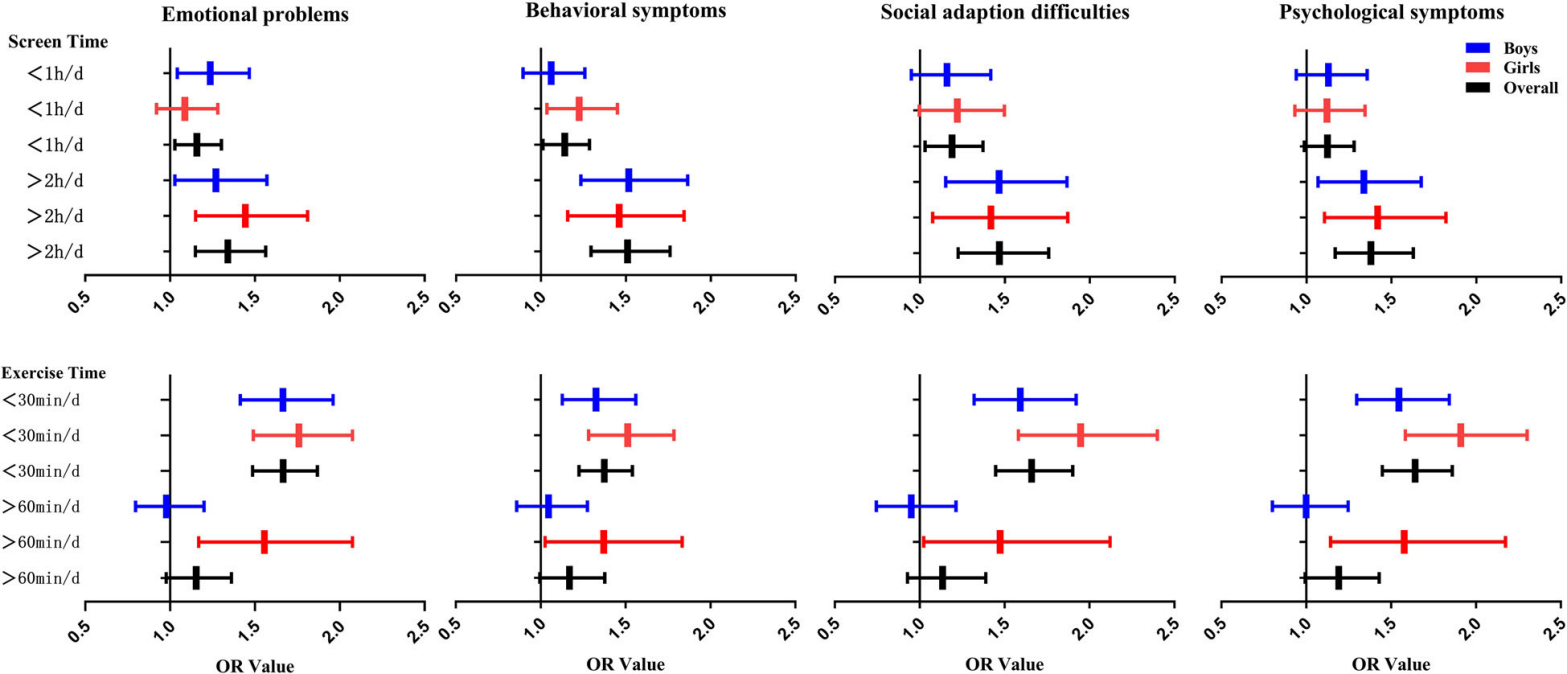
Odds Ratio Distribution from the Binary Logistic Regression

As shown in Fig. 3, a screen time > 2 h/d and an exercise time < 30 min/d were risk factors for emotional symptoms, behavioural symptoms, social adaptation difficulties, and psychological symptoms among Chinese adolescents.

The results showed that under the condition of the same screen time, < 30 min/d of exercise time was associated with the highest psychological symptoms, followed by > 60 min/d of exercise time. A combination of 30–60 min/d exercise time and 1–2 h/d screen time was the best combination for lower psychological symptoms (Table 4).

**Table 4:** Interaction of screen time and exercise time on psychological symptoms among Chinese adolescents

Classification of interaction		Emotional problems		Behavioral symptoms		Social adaption difficulties		Psychological symptoms	
		***OR***(95%***CI***)	***P***	***OR***(95%***CI***)	***P***	***OR***(95%***CI***)	***P***	***OR***(95%***CI***)	***P***
Screen time (h/d)	Exercise time (min/d)								
<1	<30	1.73 (1.45–2.07)	< 0.001	1.41 (1.18–1.68)	< 0.001	1.80 (1.45–2.24)	< 0.001	1.62 (1.33–1.97)	< 0.001
	30–60	1.13 (0.93–1.38)	0.23	1.04 (0.86–1.27)	0.68	1.06 (0.83–1.36)	0.63	1.00 (0.80–1.25)	1.00
	>60	1.43 (1.11–1.86)	0.01	1.37 (1.06–1.76)	0.02	1.25 (0.90–1.72)	0.18	1.40 (1.06–1.86)	0.02
1–2	<30	1.64 (1.34–2.02)	< 0.001	1.25 (1.02–1.54)	0.03	1.49 (1.16–1.92)	< 0.001	1.51 (1.21–1.89)	< 0.001
	30–60	1		1		1		1	
	>60	1.14 (0.86–1.51)	0.37	1.04 (0.79–1.38)	0.79	1.20 (0.85–1.69)	0.30	1.07 (0.78–1.47)	0.66
>2	<30	2.41 (1.91–3.05)	< 0.001	2.00 (1.59–2.52)	< 0.001	2.45 (1.86–3.21)	< 0.001	2.30 (1.79–2.96)	< 0.001
	30–60	1.25 (0.97–1.61)	0.09	1.37 (1.07–1.76)	0.01	1.39 (1.02–1.88)	0.04	1.25 (0.94–1.65)	0.12
	>60	1.29 (0.92–1.82)	1.14	1.63 (1.18–2.24)	< 0.001	1.33 (0.88–1.99)	0.17	1.30 (0.90–1.87)	0.17

## Discussion

The present study examined the associations among screen time, exercise time, and self-rated psychological symptoms in Chinese adolescents. We also investigated interactions between screen time and exercise time regarding the risk for psychological symptoms, after controlling for weight status, to determine the best combination of exercise time and screen time to promote the mental health of Chinese adolescents. We found that screen time and exercise time played independent roles in psychological symptoms and increased the risk for psychological symptoms through their interaction, which was consistent with the result of a previous study [39]. A combination of 1–2 h/d of screen time and 30–60 min/d of exercise time was considered the best combination for better mental health. Increasing evidence shows that excess screen time among adolescents harms mental health [40]. It was reported that Chinese urban adolescents who engaged in screen time more than 2 h/d had higher risks for depressive symptoms (OR = 1.52), anxiety symptoms (OR = 1.36), and school life dissatisfaction (OR = 2.07) than those with screen time < 2 h/d [22]. Previous studies have also suggested that adolescents with a screen time > 2 h/d have lower self-esteem [41], poorer health [42], and poorer academic performance [43]. Screen time is also considered a risk factor for Internet addiction [44]. In addition, the detection rates of emotional symptoms, behavioural symptoms, and social adaptation difficulties among adolescents with a screen time < 1 h/d were significantly higher than those among adolescents with a screen time of 1–2 h/d, which differed from previous studies [45].

With the rapid development of electronic technology and information, it is necessary and effective for adolescents to acquire knowledge about entertainment, social interaction, and health through mobile phones, television, and other media [46], which may help adolescents’ physical and mental health development [47]. Given the current development of information technology, various types of physical and mental entertainment [48] and communication [49] need to be performed using a screen. Eliminating the Internet and other screen media may cause children and adolescents to be isolated from society, thereby preventing their effective integration into society [47], which in turn would result in psychological problems. Odgers et al. showed that the appropriate use of technology, such as the Internet, to make friends online and arrange offline gatherings or exchanges between friends may help alleviate and release psychological stress and exert a positive impact on mental health [15].

Our study showed that the detection rate of psychological symptoms was lowest among adolescents with an exercise time of 30–60 min/d and highest among those with an exercise time of 30 min/d, which was a risk factor for adolescents’ mental health. Physical exercise has a protective role in preventing the occurrence of mental disorders among children and adolescents [50], and mental health is positively correlated with exercise time [51] and the amount of physical activity [52]. However, longer exercise durations are not always better. A recent study showed that exercising 30–60 min/d 3–5 days a week had the best effect on adolescents’ mental health, whereas exercising more than 90 min/d or more than 23 times a month resulted in worse mental health than not exercising [53]. A study conducted by the American Aerobic Fitness Center suggested that daily exercise, whether aerobic or anaerobic, should be controlled to durations of 40–60 min to maintain good physical and mental health [54]. In the present study, the detection rate of psychological symptoms was lowest among adolescents who exercised 30–60 min/d, whereas the detection rates of psychological symptoms among adolescents who exercised > 60 min/d or < 30 min/d were higher. Notably, we considered exercise time only, and other types of physical activity, such as active transport or housework, were not included. Therefore, we recommend that adolescents engage in daily physical activity and adhere to the reference we provide in our study.

Screen time and physical activity are independent factors rather than functional opposites [55]. However, Motl et al. confirmed a negative correlation between screen time and physical activity among adolescents [56]. Simon et al. showed that decreased screen time and increased physical activity had a positive impact on adolescents’ sense of self-efficacy and energy levels [57]. The present study showed that under conditions of the same screen time, adolescents with < 30 min/d or > 60 min/d of exercise time had more psychological symptoms than those with 30–60 min/d of exercise time. Screen time and exercise time interacted to increase the risk of psychological symptoms. However, it was unclear whether the effect of screen time on psychological symptoms was the result of decreased exercise time or increased sedentary behaviour. Sedentary behaviour itself may be an independent risk factor for psychological symptoms, and it is necessary to further explore the relationships among these factors.

This study had several strengths. The sample was representative because it was drawn from six regions of China and was well distributed. Additionally, the present study may provide a reference for adolescents to reasonably manage their screen time and exercise time. There were also some limitations to the present study. First, this study used a cross-sectional design, which prevented the identification of causal relationships. Longitudinal studies are needed to explore the relationships among screen time, exercise time, and psychological symptoms. Second, screen and exercise time were evaluated by adolescents’ self-assessment, which was influenced by adolescents’ recall ability, so the accuracy may not be high. Third, we did not measure the type of exercise or activity adolescents engaged in, and some types may be more supportive of mental health than others.

## Conclusions

In summary, there were associations between screen time, exercise time, and psychological symptoms in Chinese adolescents. Reasonable management of exercise time (e.g., 30–60 min/d) and screen time (e.g., 1–2 h/d) may play a positive role in promoting the mental health of adolescents. The present study may provide a reference for the primary and secondary prevention of mental health problems among adolescents. Research on the effects of different types of exercise and different screen times on mental health is needed.

## Data Availability

The datasets used during the current study cannot be made publicly available as per ethics approval at East China Normal University. Readers can obtain them from the corresponding author on reasonable request.

## Abbreviations

MSQA: Multidimensional Sub-health Questionnaire of Adolescents
BMI: Body mass index
OR: Odds ratio
χ^2^: Chi-square
ESRI: Environmental Systems Research Institute
